# Case of Triplets

**Published:** 1856-06

**Authors:** J. Levergood


### *Case of Triplets.* By J. LEVERGOOD, M. D.

In the February No. of the Examiner, the writer recorded a case of triplets that occurred in his practice, and noted a peculiarity in the excessive shortening of the funis, in connection with it. Since then, another triplet *accouchement* has startled our community from its propriety; and well it might, it making the third case of the kind that has occurred in this borough within the last forty-eight months. When we take into consideration the fact, that the last census gave us a population of but 1310 souls, we can still better appreciate the remarkable nature of the occurrence I have mentioned, as statistics prove triple births to be an unusual event in obstetrical practice. In James' Burns we find it stated, that "more than three are not met with once in twenty thousand times." Cazeaux says, in speaking of multiple pregnancy, "triplets are very rare, since there were but five in the records of 36,441 *accouchements* that occurred at la Maternité in Paris. On referring to Churchill's elaborate statistical tables, we there find, that in a total of 448,998 cases, there were only 77 triplets, or 1 in 5,831. Two of the three females referred to were previously confined with twins.

Through the kindness of Dr. John A. Thompson, in whose practice the case happened, I am placed in possession of such facts as are necessary to constitute a satisfactory account of it; and they are succinctly these:—

Was called Jan. 25th, to attend Mrs. P., æt. 38, in her fourth labor. A per vaginam examination discovered the os uteri tolerably well dilated, membranes unruptured, and head presenting. The pains increasing both in frequency and force, the bag of waters was ruptured, and shortly afterwards a healthy male child was born. There being evidence of another fœtus in utero, an examination detected the feet of a second child, and its delivery speedily followed. This was a female, and lived but a few hours after its birth. Upon the *touch* being again applied, a third child was found, head presenting, and its delivery was not long delayed, it being a vigorous male infant. Slight traction upon the cords had the effect to bring away three separate placentæ, and the parturition was completed in two hours from the commencement of the parturient throes.

